# *MORG1*^+/−^ mice are protected from histological renal damage and inflammation in a murine model of endotoxemia

**DOI:** 10.1186/s12882-018-0826-4

**Published:** 2018-02-05

**Authors:** Tzvetanka Bondeva, Claudia Schindler, Katrin Schindler, Gunter Wolf

**Affiliations:** 1 Department of Internal Medicine III, Jena University Hospital, Am Klinikum 1, D-07740 Jena, Germany; 2 Centre for Sepsis Control and Care (CSCC), Jena University Hospital, Jena, Germany

**Keywords:** Endotoxemia, MORG1, PHD3, HIF-2α, Renal injury, Inflammation

## Abstract

**Background:**

The MAPK-organizer 1 (MORG1) play a scaffold function in the MAPK and/or the PHD3 signalling paths. Recently, we reported that *MORG1*^+/−^ mice are protected from renal injury induced by systemic hypoxia and acute renal ischemia-reperfusion injury via increased hypoxia-inducible factors (HIFs). Here, we explore whether *MORG1* heterozygosity could attenuate renal injury in a murine model of lipopolysaccharide (LPS) induced endotoxemia.

**Methods:**

Endotoxemia was induced in mice by an intraperitoneal (i.p) application of 5 mg/kg BW LPS. The renal damage was estimated by periodic acid Schiff’s staining; renal injury was evaluated by detection of urinary and plasma levels of neutrophil gelatinase-associated lipocalin and albumin/creatinine ratio via ELISAs. Renal mRNA expression was assessed by real-time PCR, whereas the protein expression was determined by immunohistochemistry or Western blotting.

**Results:**

LPS administration increased tubular injury, microalbuminuria, IL-6 plasma levels and renal TNF-α expression in *MORG1*^*+/+*^ mice. This was accompanied with enhanced infiltration of the inflammatory T-cells in renal tissue and activation of the NF-κB transcription factors. In contrast, endotoxemic *MORG1*^*+/−*^ showed significantly less tubular injury, reduced plasma IL-6 levels, significantly decreased renal TNF-α expression and T-cells infiltration. In support, the renal levels of activated caspase-3 were lower in endotoxemic *MORG1*^*+/−*^ mice compared with endotoxemic *MORG1*^*+/+*^ mice. Interestingly, LPS application induced a significantly higher accumulation of renal HIF-2α in the kidneys of *MORG1*^+/−^ mice than in wild-type mice, accompanied with a diminished phosphorylation of IκB-α and IKK α,β and decreased *iNOS* mRNA in the renal tissues of the LPS-challenged *MORG1*^+/−^ mice, indicating an inhibition of the NF-κB transcriptional activation.

**Conclusions:**

*MORG1* heterozygosity protects against histological renal damage and shows anti-inflammatory effects in a murine endotoxemia model through modulation of HIF-2α stabilisation and/or simultaneous inhibition of the NF-κB signalling. Here, we show for the first time that MORG1 scaffold could represent the missing link between innate immunity and inflammation.

**Electronic supplementary material:**

The online version of this article (10.1186/s12882-018-0826-4) contains supplementary material, which is available to authorized users.

## Background

Sepsis is a systemic response of the body to host infections. Septic conditions are often clinically difficult to diagnose and contribute to higher morbidity and mortality rates in ICUs world-wide [1]. Increased mortality due to sepsis is oft caused by bacterial infections and the lipid A-containing lipopolysaccharide (LPS), a component of the bacterial wall of the gram-negative bacteria, is a major player in the pathogenesis the of gram-negative sepsis [2]. The application of animal models have demonstrated that LPS-induced endotoxemia provokes tissue inflammation by a massive release of the inflammatory mediators as inflammatory cytokines and can lead to acute kidney injury in mice [3]. LPS treatment induced in animal models caused a systemic hypotension and organ dysfunction similar to what occurs in clinical sepsis [4], thus providing insight into the understanding of the pathogenesis of the acute renal injury. Recent studies have shown that LPS can activate the hypoxia-inducible transcription factor-1 alpha (HIF-1α) under normoxic conditions [5]. The pharmacological activation of HIF-1α through inhibition of HIF-prolyl-hydroxylases (PHDs) [6] or administration of erythropoietin, a HIFs target gene, improved renal function and reduced acute kidney injury in endotoxemic mice [7] as well as in mice suffering from polymicrobial sepsis [8]. Recently, we reported that preconditional suppression of PHDs by application of 3,4-dihydroxybezoate (3,4-DHB), a non-specific PHDs inhibitor, was renoprotective in two murine septic models [9]. However, this effect was mainly localised to the kidney, we did not observe a protective systemic effect in the survival study [10]. The discovery of the MAPK-organizer 1 (MORG1), a molecular scaffold for multiple proteins of the MAPK cascade [11] and a binding partner of the HIF-prolyl hydroxylase domain containing protein 3 (PHD3) [11] opens a new opportunity to study the role of pre-elevated HIFs level and its function in AKI due to sepsis and inflammation. Genetic manipulation of *MORG1* expression has revealed that *MORG1*^*−/−*^ mice exhibit embryonic lethality, whereas *MORG1*^*+/−*^ mice did not show phenotypic differences with the wild-type mice. Moreover, we found that *MORG1*^*+/−*^ animals are protected from renal injury in a murine model of ischemia/reperfusion due to increased HIF-1,2α expression and stabilisation [12]. In normoxia HIFs are hydroxylated by PHDs followed by ubiquitination and proteasomal degradation [13]. Reduced oxygen supply in the cells is detected by PHDs and this inhibits their HIF-prolylhydroxylase activity, resulting in HIF stabilisation and transcriptional activation. Recent research from our lab has also shown that reduced expression of MORG1 could contribute to cellular adaptation to ischemic/ hypoxic conditions through the cellular binding partner(s) PHD3/HIFs. In a murine hypoxia model *MORG1*^*+/−*^ mice were protected from systemic hypoxia- dependent renal injury due to an enhanced stability of HIF-1,2α and/or a reduced TNF-α expression in a PHD3/MORG1 dependent manner [14] compared to the *MORG1*^*+/+*^ mice which developed overt renal damage and inflammation in an animal model of systemic hypoxia [14]. Thus, we hypothesised that *MORG1* heterozygosity could attenuate kidney damage and inflammation, thus representing an important tool to gain insight into the cellular mechanisms of renal injury and inflammation related hypoxia in a well-established murine model of LPS-induced endotoxemia.

## Methods

### Animal treatment and endotoxemia induction

The animal experiments were performed according to the guidelines set by the local Animal Committee of the State of Thuringia application (file numbers 02–023/10 and 02–023/11). The animal experiments were approved by the Animal Committee of the State of Thuringia (Thueringer Landesamt fuer Lebensmittelsicherheits und Verbraucherschutz Abt. 2, Gesundheitlicher Verbraucherschutz, Veterinaerwesen, Pharmazie, Bad Langensalza, Germany) with files numbers 02–023/10 and 02–023/11 and were carried out in accordance to the National Institute of Health Guidelines for the Care and Use of Laboratory Animals (8th edition; available online: https://www.ncbi.nlm.nih.gov/books/NBK54050/) and to the European Community Council Directive for the Care and Use of Laboratory Animals (Directive 2010/63/EU; http://ec.europa.eu/environment/chemicals/lab_animals/legislation_en.htm). The study was performed on wild-type mice C57BL/6 J (Jackson Laboratories, Main, USA obtained from Charles River Laboratories, Sulzfeld, Germany) and *MORG1* heterozygous animals. The *MORG1*^*+/−*^ mice were generated as described in [12] and were backcrossed for more than 12 generation to mice with C57BL/6 J genetic background. 12–16 weeks old *MORG1*^*+/+*^ and *MORG1*^*+/−*^ male mice, weighting 20–25 g were used in the study and received a standard diet and free access to tap water. 18 wild-type mice (*MORG1*^*+/+*^) and 18 heterozygous *MORG1* (*MORG1*^*+/−*^) animals were used for the study and were randomly separated into 4 groups (*n* = 9 per group) see below. All animals were bred and maintained at the “Central Experimental Animal Facilities (ZET)” at Friedrich Schiller University Hospital, Jena, Germany with regular 12/12 h light/ dark cycles and 23 ± 1 °C room temperature. The mice used in the experimental procedure were age and sex matched and were randomly separated into the experimental groups and either received intraperitoneal injection (i.p.) of 0.9% NaCl (*MORG1*^*+/+*^ and *MORG1*^*+/−*^ control group) for 24 h or an i.p. application of 5 mg/kg BW lipopolysaccharides from *E. coli* O111:B4 (LPS) to induce endotoxemia, purchased from Sigma-Aldrich Chemie GmbH, (Taufkirchen, Germany) for 24 h (*MORG1*^*+/+*^ and *MORG1*^*+/−*^ LPS group). The experimental treatments were performed according to the approved experimental LPS dose and procedure from the Animal Committee of the State of Thuringia, Bad Langensalza, Germany (file 02–023/10). Briefly, the LPS dose of 5 mg/kg BW was freshly prepared and injected once by i.p. application + a vehicle solution of 25 μl/ g BW of 0.9% NaCl, which was applied by subcutaneous injection. The treatments were performed under a short isofluran anaesthesia using a standard procedure described elsewhere [15–17]. The clinical status of the animals was evaluated every 4 h by applying a Clinical Severity Score (CSS) as described previously [18]. The score uses the following parameters and ranges from 1 to 4 for each of them: spontaneous activity; reaction to exogenous stimuli and posture [18]. 24 h after treatments the mice were deep anesthetised with isofluran before being sacrificed by cervical dislocation as approved by the Animal Committee of the State of Thuringia and described in the European Community Council Directive for the Care and Use of Laboratory Animals (Directive 2010/63/EU; ANNEX IV (3.Table).The kidneys were collected and paraffin embedded or kept frozen at − 80 °C for further analysis. Blood samples were collected for blood plasma isolation and analysis. The collected urine samples were also stored at − 80 °C until analysed.

### Survival analysis

The systemic effect of endotoxemia was investigated by survival studies as described previously [15]. The survival study was approved from the Animal Committee of the State of Thuringia (file number 02–023/11). The survival analyses were carried out because there are so far no data about the *MORG1*^*+/−*^ mice sensitivity to lipopolysaccharide exposure. The mice were randomly separated into experimental groups as described above. Each group contained 10 animals and received a single dose of 0.9% NaCl or LPS. The LPS application was performed as described above. During the 72 h survival analyses the mice were weighed once daily and the clinical well-being of the animals was estimated by the application of CSS [18], which was evaluated every 4 h. The CSS was approved by the Animal Committee of the State of Thuringia. Subsequently the animal’s survival was monitored and recorded every 6 h over a 72 h period and the mice had a free access to water and standard rodent food. The animals who survived the analyses were deep anesthetised with isofluran before being sacrificed by cervical dislocation, a standard procedure, in accordance to the approved protocol by the Animal Committee of the State of Thuringia (file number 02–023/11).

### Evaluation of kidney function and morphology

The kidney functional parameter or renal morphology and tubular injury were performed on kidney harvested 24 h post induction of endotoxemia by LPS administration or application of saline solution in the corresponding experimental groups. In order to calculate the urinary albumin-creatinine-ratio (ACR) during the corresponding treatment, the animals were kept in metabolic cages (Techniplast, Hohenpeißenberg, Germany) and 24 h urine was collected. The urinary levels of creatinine and albumin were determined by enzyme-linked immunosorbent assay (ELISA), respectively from Cayman Chemical Company (Tallinn, Estland) and CellTrend GmbH (Luckenwalde, Germany). The urinary concentration of neutrophil gelatinase-associated lipocalin (NGAL) of the corresponding treatment, we also measured in 24 h collected urine. The urinary and plasma levels of NGAL were detected by mouse NGAL ELISA kit, purchased from BioPorto Diagnostics, Gentofte, Denmark. The assay was performed according to manufacturer instructions. The limit of the sample detection was 75 pg/ml and the assay range was 10–1000 pg/ml NGAL. The plasma levels of creatinine (Cre) and blood urea nitrogen (BUN) were measured in blood plasma 24 h post LPS application or saline injection in all experimental groups by colorimetric chip assay on clinical chemical analyzer (Fuji DRI-CHEM 3500i, Fujifilm, Dusseldorf, Germany). The Cre and BUN concentrations are expressed in mg/dl. Tubular damage was estimated by periodic acid Schiff-reaction (PAS) performed on 2 μm paraffin kidney sections using a PAS staining kit (Baacklab, Schwerin, Germany). The staining was evaluated using an Axioplan microscope and AxioVision Rel. 4.6. software (Zeiss, Jena, Germany). The tubular damage was estimated by scoring system where 0 represent no damage and 5 corresponded to more than 90% injured proximal tubule. We also investigated the proximal tubular cells injury based on the positivity of immunohistological detection of Kidney injury molecule 1 (KIM1) in renal tissue in all experimental groups. The number of damaged (positively stained) tubuli per kidney section was counted and the average number is graphically presented.

### Assessment of protein expression in renal tissue by immunohistochemistry

The kidneys were fixed in 10% neutral-buffered formalin and were paraffin embedded. For immunohistological analyses, we used 4 μm paraffin kidney sections with heat-induced antigen retrieval, as described elsewhere [19]. For immunohistochemistry the kidney sections were blocked with 5% BSA for 1 h at room temperature, followed by incubation with the primary antibodies overnight at 4 °C. The following primary antibodies were used: goat-polyclonal antibody anti-HIF-2α purchased from R&D Systems (Wiesbaden, Germany) and used in 1:200 dilution, an anti-CD3 antibody (1:100 dilution) was obtained from Dianova GmbH (Hamburg, Germany), an anti-PHD3 antibody (1:100 dilution) was from Santa Cruz Biotechnology (Heidelberg, Germany), and anti- KIM1 antibody (1:1000 dilution) was purchased from Cloud One Corp. (Houston, TX, USA). The corresponding secondary antibodies, HRP-conjugated (1:500 dilution), were purchased from KPL Inc. (Gaithersburg, MD, USA). Peroxidase substrates were either 3-amino-9-ethylcarbazole (AEC) or diaminobenzidine (DAB) as appropriate, both purchased from (Vector Laboratories Inc., Burlingame, CA, USA). When appropriate the nuclei were counter-stained with hematoxylin (Vector Laboratories Inc.) for 2 min. The stains were mounted and microscopic analyses were performed by Axioplan microscope and AxioVision Rel. 4.6. software (Zeiss, Jena, Germany). A minimum of 4–6 animals per experimental group were investigated. Routinely, the staining was analysed “blind” from a person unaware of the experimental protocol via a semi-quantitative scoring method (for detection of CD3 positive cells) to estimate the staining intensity as previously reported [20] or the number of detectable positively stained cells per field were counted by Image software using a cell counting analyses and presented graphically as mean ± SEM.

### Assessment of protein expression in renal tissue by western blot analyses

For protein analyses the kidneys were extracted 24 h following LPS i.p. application or saline solution and the protein expression was evaluated. Assessment of protein was performed in kidneys homogenised in complete lysis M supplemented with inhibitors (Roche, Mannheim, Germany) and 1 mM sodium-orthovanadate. Routinely 3 to 4 animals per group were randomly selected for Western blot analyses. The lysates were vortexed, kept on ice for 15 min and centrifuged at 14,000 rpm for 20 min at 4 °C. The supernatant was then used for the further analysis. To evaluate the protein expression 12% or 15% SDS-PAGE were performed, followed by transfer of the gels onto a PVDF membrane. The detection of the protein was performed by ImageQuantTM LAS 4000 biomolecular imager system (GE Healthcare, Upsala, Sweden) via ECL- visualisation. The following antibodies were used for protein detection: anti-phospho Iκ-Bα (1: 200 dilution), anti- Iκ-Bα (1: 200 dilution), anti-phospho-IKKα,β (Ser 180, Ser181) (1: 200), anti-IKKα (1: 200), anti-vinculin (1: 2000 dilution), anti-TNFα (1: 200 dilution), anti-PCNA (proliferating cell nuclear antigen) (1: 500 dilution), anti-NF-κB (1:200). All antibodies were purchased from Santa Cruz Biotechnology (Heidelberg, Germany). The expression of caspase-3 was detected by anti-caspase-3 antibody (1:1000 dilution) purchased from abcam, Cambridge, UK. The assessment of the nuclear NF-κB levels was performed by preparation of kidney nuclear extracts by the use of ProteoExtract® Subcellular Proteome Extraction kit (Merck, Darmstadt, Germany). The expression of the proteins was subjected to densitometry measurement using ImageJ software.

### RNA isolation, reverse transcription and real - time PCR

Renal expression of the genes of interest was analysed 24 h post endotoxin administration or saline injection. Renal tissue was homogenized using a SpeedMill P12 homogenizer (Analytik Jena Bio Solutions, Jena, Germany) and total RNA was isolated using the RNeasy kit (Qiagen, Hilden, Germany) according to the manufacturer’s instructions. The synthesis of cDNA was performed routinely from 1 μg of total RNA using the M-MLV Reverse Transcription system (Invitrogen Life Technologies, Darmstadt, Germany). The gene specific primers for the real – time PCR (RT-PCR) are as follows: *tnf-α* - forward: 5’-GGCAGGTCTACTTTGGAGTCATTGC-3′, reverse: 5’ ACATTCGAGGCTCCAGTGAATTCGG 3′, *hprt (hypoxanthine phosphoribosyltransferase)* forward: 5’-ATCAGTCAACGGGGGACATA-3′, reverse: 5’-AGAGGTCCTTTTCACCAGCA-3′, *hif-2α* - forward: 5’-AAGCTCCTGTCCTCAGTCTG-3′, *hif-2α* reverse: 5’-CATCCTCATGAAGAAGTCAC-3′, *pdh3* forward: 5’-GCTATCCAGGAAATGGGACA-3′, *pdh3* reverse: 5’-GGCTGGACTTCATGTGGATT-3′ *ngal (lipocalin-2)* forward: 5’-CACCACGGACTACAAGTTCGC-3′, 3′ *ngal (lipocalin-2)* reverse: 5’-TCAGTTGTCAATGCATTGGTCGGTG-3′, *Epo* forward: 5-CCACCCTGCTGCTTTTACTC-3′, *Epo* reverse: 5’-CTTGAAGAGAACCTGGGAGT-3′, *iNOS (inducible nitric oxide synthase, also known as NOS2)* forward: 5’ AGCTGGCTCGCTTTGCCACG 3′, *iNOS* reverse: 5’ GCCTCCTTTGAGCCCTTTGT 3′; *Kim1* (Kidney injury molecule 1) forward: 5’ ATGAATCAGATTCAAGTCTTC 3′, *Kim1* reverse: 5’ TCTGGTTTGTGAGTCCATGTG 3′. All primers were purchased from Invitrogen, Darmstadt, Germany. The relative expression of the gene of interest was quantified by RT-PCR using a Q-tower thermocycler (Analytik Jena Bio Solutions, Jena, Germany). The quantitative real-time PCR was done as previously described The expression of the gene of interest was normalized to the expression of *hprt* and the relative expression ratio was quantified by ΔΔCT method, where *R* = 2^-ΔΔCT^ [21]. The mRNA levels in saline treated *MORG1*^*+/+*^ mice was set as 1.

### Statistical analysis

All values are presented as mean ± standard error of mean (SEM). Statistical analyses were performed with the statistical package SigmaPlot 13 software (SYSTAT Software, San Jose, CA, USA). Results were evaluated by the Kruskal-Wallis One way Analyses of Variances on Ranks test, followed by the Mann-Whitney-Rank Sum-Test to analyse the differences between two groups. All Pairwise Multiple Comparison Procedures were performed with Student-Newman-Keuls Method. Survival was analysed by the Kaplan-Meier test with log-rank statistic. Differences were considered significant when *p* < 0.05.

## Results

### Reduced MORG1 expression helped prevent damage to renal tissue after administration of LPS

**Fig. 1:**
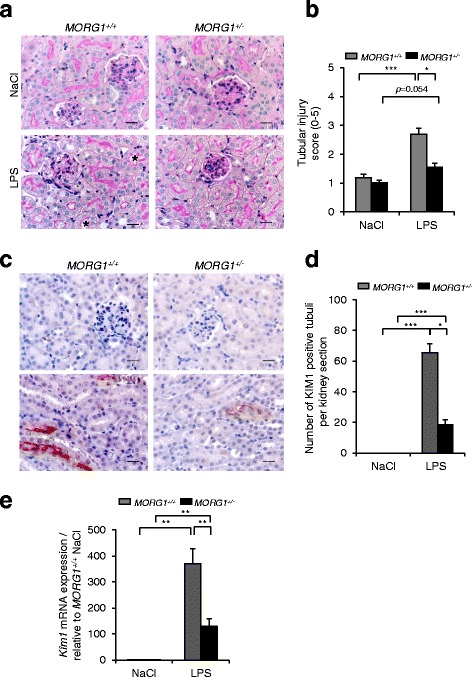
**a-e**: Analysis of renal damage. **a** Detection of renal injury by PAS- staining 24 h post LPS administration. LPS treatment induced cortical tubular dilatation in *MORG1*^*+/+*^ mice. Representative images are shown. The vasodilation and brush border damages are shown with asterisk on the images. Original magnification 400×. Bars correspond to 20 μm. *N* = 6 per group. **b** Semi-quantitative cortical tubular injury scores. The score ranges from 0 to 5 depending on level of tubular damage. N = 6, *MORG1*^*+/+*^/LPS vs. *MORG1*^*+/+*^ saline treated mice, *p* < 0.001; *MORG1*^*+/+*^/LPS vs. *MORG1*^*+/−*^/LPS, *p =* 0.017. *p < 0.05, ***p < 0.001. **c** Detection of the tubular damage by immunohistochemical detection of the Kidney injury molecule 1 (KIM1) protein expression in renal sections 24 h post endotoxemia or saline treatment. Representative images are shown. Original magnification 400×. Bars 20 μm. *N* = 4. **d** Number of KIM1 positive tubuli per kidney section are graphically presented. N = 4 mice per group. *MORG1*^*+/+*^/LPS versus *MORG1*^*+/−*^/LPS, *p =* 0.029, *p < 0.05. *MORG1*^*+/+*^/LPS vs. *MORG1*^*+/+*^*/*saline*,* ***p < 0.001. *MORG1*^*+/−*^/LPS vs. *MORG1*^*+/−*^/saline, ***p < 0.001. **e** Real-time PCR analyses of the *Kim1* mRNA expression in endotoxemic and saline treated wild-type and MORG1^*+/−*^ mice. LPS induced a robust increase in the *Kim1* mRNA expression in both genotypes. Nevertheless, a down-regulation of MORG1 was associated with a significantly lower expression of *Kim1* compared with the wild-type endotoxemic mice. N = 4 *MORG1*^*+/+*^*/* NaCl; *N* = 8 *MORG1*^*+/+*^/LPS; *N* = 7 *MORG1*^*+/−*^/NaCl; *N* = 5 *MORG1*^*+/−*^/LPS. *MORG1*^*+/+*^/LPS vs. *MORG1*^*+/−*^/LPS *p =* 0.0097, **p < 0.01

It is well documented that endotoxemia causes tubular damage in murine models [22]. To assess the LPS effect on renal tissue we performed periodic acid Schiff’s (PAS) staining on kidney paraffin sections. We detected a significantly (*p* < 0.001) increased tissue damage (score 2.7 ± 0.198) most prominently in the cortex, manifested by tubular dilation and vacuolisation (shown with asterisk on the images) in wild-type *MORG1*^*+/+*^ mice due to LPS administration compared with the saline treated wild-type mice (score 1.19 ± 0.115) (*p* < 0.001) (Fig. 1a, b). However, endotoxemic heterozygous *MORG1*^*+/−*^ mice showed significantly (*p* = 0.017) less dilation of proximal tubules compared with LPS treated *MORG1*^*+/+*^ mice (Fig. 1a, b). We also analysed the expression of KIM1 (Kidney injury molecule 1) in the renal sections as a marker of the early tubular damage [23, 24]. As expected in saline treated wild-type and heterozygous *MORG1*^*+/−*^ mice, expression of KIM1 was not found, while LPS administration significantly induced the protein (Fig. 1c, d). The injured proximal tubuli showed a positive immunological stain of KIM1, localised to the apical brush border of the proximal tubular epithelial cells (Fig. 1c). We counted the number of KIM1 positive tubuli per kidney section and found that endotoxemic *MORG1*^*+/−*^ mice had 18.75 ± 2.87 injured proximal tubuli compared with their LPS treated wild-type littermates 65.5 ± 5.95, (*p* = 0.029). Immunohistochemistry revealed that KIM1 tubular expression was induced in both genotypes upon LPS treatment, nevertheless we detected significantly more KIM1 positive tubuli in endotoxemic wild-type *MORG1*^*+/+*^ compared with the *MORG1*^*+/−*^ LPS treated mice (Fig. 1c, d). We next assed the *Kim1* mRNA in renal tissue and found that endotoxemia induced *Kim1* mRNA expression was significantly augmented in both genotypes compared with saline treated controls *p* = 0.004 and *p* = 0.003 for *MORG1*^*+/+*^ /LPS and *MORG1*^*+/−*^ /LPS mice, respectively. Moreover*, MORG1* heterozygosity decreased significantly the *Kim1* mRNA levels (128.96 ± 29.81 fold) then the corresponding endotoxemic wild-type littermate (369.87 ± 57.36 fold), (*p* = 0.003) (Fig. 1e).

### MORG1 suppression reduced renal NGAL expression in endotoxemic animals

**Fig. 2:**
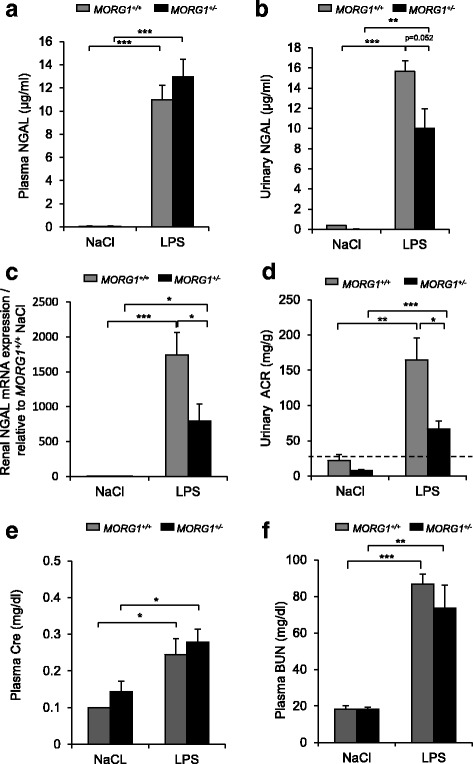
**a-f**: Analysis of renal functional parameters 24 h following endotoxemia induction or saline solution application. **a** Determination of the plasma concentration of Neutrophil gelatinase associated lipocaline (NGAL). *N* = 6 per group, ***p < 0.001. **b** Measurement of the NGAL urine concentration in 24 h collected urine. At least 6 animals per group were subjected to the analysis. *MORG1*^*+/−*^ LPS vs. *MORG1*^*+/−*^ NaCl treated mice, *p* = 0.002; *MORG1*^*+/+*^ LPS vs. *MORG1*^*+/+*^ NaCl treated mice, *p* < 0.001. **p < 0.01, ***p < 0.001. **c** Determination of the renal NGAL mRNA expression via real-time PCR analyses. The expression is presented as a ratio relative to the saline treated *MORG1*^*+/+*^ mice. N = 4 per group, *MORG1*^*+/+*^ LPS vs. *MORG1*^*+/−*^ LPS, *p* = 0.021; *MORG1*^*+/−*^ LPS vs. *MORG1*^*+/−*^ NaCl treated mice *p* = 0.049; *MORG1*^*+/+*^ LPS vs. *MORG1*^*+/+*^ NaCl treated mice, *p* < 0.001. *p < 0.05, ***p < 0.001. **d** Detection of renal injury by determination of urinary ACR (uACR). LPS treatment significantly increased the uACR in *MORG1*^*+/+*^ and *MORG1*^*+/−*^ mice compared with the corresponding controls, *p* = 0.008 and *p* < 0.001 respectively; the levels of uACR are significantly lower in endotoxemic *MORG1*^*+/−*^ mice relatively to the equally treated *MORG1*^*+/+*^ mice, *p* = 0.03. *N* = 6 per group. *p < 0.05, **p < 0.01, ***p < 0.001. **e** Detection of plasma creatinine (Cre) levels 24 h following endotoxemia or saline application. N = 7 per group for *MORG1*^*+/+*^ saline and *MORG1*^*+/−*^ saline, *N* = 9 mice per group for *MORG1*^*+/+*^ LPS and *MORG1*^*+/−*^ LPS. LPS induced increase in plasma Cre in both genotypes compared with the control saline treated groups. *MORG1*^*+/+*^ LPS vs. *MORG1*^*+/+*^ saline treated mice, *p* = 0.012, *MORG1*^*+/−*^ LPS vs. *MORG1*^*+/−*^saline *p = 0.0156, *p < 0.05. **f** Detection of plasma blood urea nitrogen (BUN) concentrations 24 h following endotoxemia or saline application. N = 7 per group for *MORG1*^*+/+*^ saline and *MORG1*^*+/−*^ saline, N = 9 mice per group for *MORG1*^*+/+*^ LPS and *MORG1*^*+/−*^ LPS. Endotoxemia elevated the plasma BUN concentrations in both genotypes compared with the control saline treated groups. *MORG1*^*+/+*^ LPS vs. *MORG1*^*+/+*^ saline treated mice, *p* = 0.001, *MORG1*^*+/−*^ LPS vs. *MORG1*^*+/−*^saline *p* = 0.004, **p < 0.01, **p < 0.001

We also investigated acute renal injury by determining the urinary and plasma concentrations of neutrophil gelatinase-associated lipocalin (NGAL), an early marker of AKI, in wild-type and heterozygous mice (Fig. 2). As expected we found a significantly (*p* < 0.001) increased NGAL levels in plasma due to the LPS application, in both mice genotype (Fig. 2a). Analyses of the urinary NGAL protein concentrations showed significantly higher levels in LPS treated wild-type and *MORG1* heterozygous mice compared with untreated controls in both genotypes, *p* < 0.001 and *p* = 0.002, for *MORG1*^*+/+*^/ LPS and *MORG1*^*+/−*^/ LPS mice, respectively. Nevertheless, the urinary concentrations of NGAL in LPS treated *MORG1*^*+/−*^ animals were reduced with about 44% (10.029 ± 1.948 μg/ml) compared with the endotoxemic wild-type mice (15.643 ± 1.074 μg/ml), although, the difference did not rich yet a statistical significance (*p* = 0.052) (Fig. 2b). Next, we investigated the renal *ngal* gene expression in wild-type and Morg1 heterozygous mice by real-time PCR. We found that the untreated mice showed extremely low levels of *nga*l mRNA, while endotoxemia sharply induced rena*l ngal e*xpression levels in both genotypes. *P* values were correspondingly *p* < 0.001 and *p* = 0.002 for *MORG1*^*+/+*^/LPS and *MORG1*^*+/−*^*/*LPS mice. Interestingly, we measured a significantly *(p* = 0.021) diminished *nga*l gene expression in *MORG1*^*+/−*^/ LPS mice (795.070 ± 76.054 fold increase) compared with *MORG1*^*+/+*^/ LPS treated mice (1742.007 ± 324.135 fold increase) (Fig. 2c). We also investigated renal function by determining the urinary albumin-creatinine-ratio (uACR) in endotoxic wild-type and heterozygous mice. The exposure to LPS induced microalbuminuria (uACR> 30 mg/g) in both genotypes, but the *MORG1*^*+/−*^ animals showed significantly (*p* < 0.05) lower uACR compared with *MORG1*^*+/+*^ mice upon LPS exposure (Fig. 2d). On the other hand, analyses of the plasma creatinine (Cre) (Fig. 2e) and blood urea nitrogen (BUN) (Fig. 2f) concentrations often used as well a marker of the renal function [24, 25] depicted that endotoxin application elevated Cre and BUN levels in both genotypes and the *MORG1* reduced expression did not significantly influence their concentrations (Fig. 2e, f).

### Endotoxemia reduced the renal expression of PHD3

**Fig. 3:**
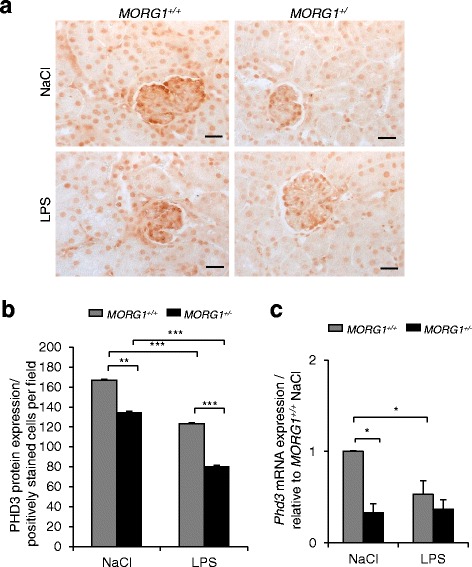
**a-c**: PHD3 renal expression in *MORG1*^*+/+*^ and *MORG1*^*+/−*^ mice 24 h after LPS or NaCl application. **a** Immunohistological detection of renal PHD3 protein expression. Representative images are shown. Original magnification 400×**.** Bars correspond to 20 μm. **b** Evaluation of the PHD3 renal immunohistology. The number of the positively stained PHD3 cells per field was counted and is graphically presented. *N* = 4 mice per treatment, *MORG1*^*+/−*^ saline vs. *MORG1*^*+/+*^ saline treated mice, *p* = 0.006; *MORG1*^*+/−*^ LPS vs. *MORG1*^*+/+*^ LPS treated mice, *p* < 0.001; *MORG1*^*+/−*^ LPS vs. *MORG1*^*+/−*^ NaCl treated mice, *p* < 0.001; *MORG1*^*+/+*^ LPS vs. *MORG1*^*+/+*^ saline treated mice, *p* < 0.001. **p < 0.01, ***p < 0.001. **c** Renal *Phd3* mRNA expression. Real-time PCR assay revealed that *MORG1* down-regulation is associated with a reduced basic expression of *Phd3* mRNA. *p < 0.05. LPS administration reduced *Phd3* expression in *MORG1*^*+/+*^ mice but did not significantly affect *Phd3* expression in *MORG1* heterozygous mice. *N* = 6 per group, *p < 0.05

Septic inflammation contributes to reduced oxygen supply and tissue hypoxia. The main cellular sensor to oxygen supply is the family of enzymes named HIF-prolyl-hydroxylases (PHDs) [13]. The MORG1 protein forms a complex with the PHD3 isoform, therefore we investigated whether the expression levels of PHD3 in renal tissue after LPS administration may be dependent on *MORG1* reduction. Immunohistology of kidney sections showed an increased PHD3 protein expression in saline treated *MORG1*^*+/+*^ compared with the *MORG1*^*+/−*^ mice (*p* = 0.006) (Fig. 3a). Furthermore, LPS treatment significantly (*p* < 0.001) reduced PHD3 expression compared with the saline treated mice in both *MORG1*^*+/+*^ and *MORG1*^*+/−*^ mice (Fig. 3a, b). In addition, the PHD3 positive cells per field were significantly lower (*p* < 0.001) in the endotoxemic *MORG1*^*+/−*^ mice (80.67 ± 3.89) compared with *MORG1*^*+/+*^ LPS treated mice (123.53 ± 4.4) (Fig. 3a, b). Real-time PCR analyses of *Phd3* mRNA expression depicted a reduced basal *Phd3* gene expression in *MORG1*^*+/−*^ mice compared to *MORG1*^*+/+*^ mice (Fig. 3c). On the other hand, LPS administration significantly (*p* < 0.05) inhibited the *Phd3* mRNA expression in the *MORG1*^*+/+*^ mice, whereas we did not find significant differences in the *Phd3* gene expressions between the LPS- treated and untreated *MORG1*^*+/−*^ mice (Fig. 3c).

### LPS administration increased renal expression of HIF-2α

**Fig. 4:**
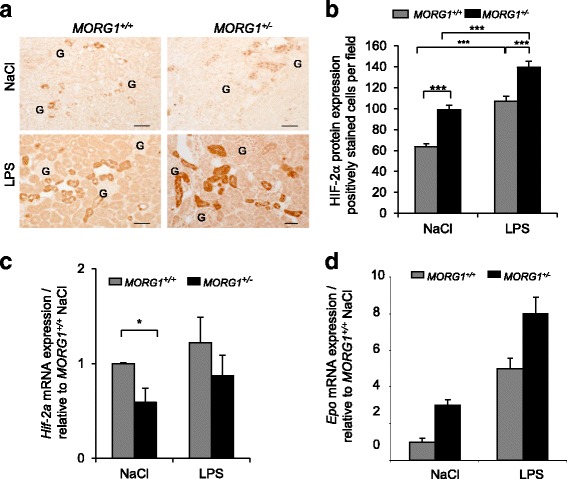
**a-d**: HIF-2α renal expression in *MORG1*^*+/+*^ and *MORG1*^*+/−*^ mice treated with LPS. **a** Protein expression of HIF-2α in kidney sections. Reduction of *MORG1* expression increased basal and LPS dependent on HIF-2α tubular/peritubular protein expression. Representative images are shown. Original magnification 200×. Bars correspond to 50 μm. G - glomerulus. **b** HIF2-α renal immunohistology evaluation. The number of the positively stained HIF2-α cells per field was counted and is graphically presented. *N* = 6 per group, *MORG1*^*+/+*^ saline vs. *MORG1*^*+/−*^ saline treated mice, *p <* 0.001; for other pairwise comparison as shown on the graph the significance was *p* < 0.001. ***p < 0.001. **c** Determination of renal *Hif-2α* mRNA expression using real-time PCR analyses. N = 6 per group, *MORG1*^*+/+*^ NaCl vs. *MORG1*^*+/−*^ NaCl, *p* = 0.018. *p < 0.05. **d** Real-time PCR analyses of erythropoietin (*Epo*) kidney mRNA expression during endotoxemia. The renal expression of *Epo* was analysed as a representative HIF-target gene. The basal levels of *Epo* were slightly, but not significantly increased in *MORG1*^*+/−*^ mice. N = 6 per group

Endotoxemia increases the HIFs activation under normoxic conditions [26]. It has been shown that PHDs differentially regulate HIFs and PHD2 and 3 isoforms are mostly involved in the prolyl-hydroxylation of HIF2-α [27, 28]. Thus, we next investigated the expression of HIF-2α in kidney sections after LPS administration. Immunohistological studies revealed that in kidney sections of *MORG1*^*+/−*^ heterozygous animals was a higher number of HIF2-α positive cells per field (99.1 ± 3.97) compared with the wild-type mice (63.5 ± 2.99), (*p* < 0.001). LPS injection further elevated the number of the HIF2-α positive cells per field in renal sections in both *MORG1*^*+/+*^ (107.1 ± 4.67) and *MORG1*^*+/−*^ (139.6 ± 5.89) mice relatively to the saline treated controls (*p* < 0.001) (Fig. 4a, b). Analyses of the gene expression of *Hif2-α* depicted reduced (*p* = 0.018) basal levels in *MORG1*^*+/−*^ relatively to *MORG1*^*+/+*^ mice (Fig. 4c). We further evaluated the renal gene expression of the mainly HIF2-α regulated gene erythropoietin in whole renal homogenates and found that its basal and LPS-dependent induction was numerically increased in the *MORG1* heterozygous animals compared to the *MORG1*^*+/+*^ mice, but did not reach statistical significance (Fig. 4d).

### Heterozygous MORG1^+/−^ deletion is associated with reduced plasma IL-6 levels in LPS treated mice

**Fig. 5:**
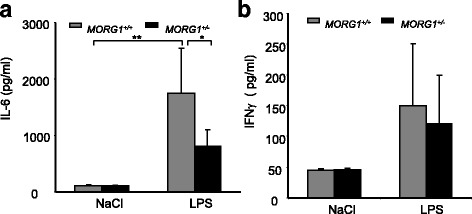
**a + b**: Influence of LPS 24 h post endotoxemia on the blood concentrations of IL-6 and IFNγ in *MORG1*^*+/+*^ and *MORG1*^*+/−*^ mice. **a** Plasma concentration of IL-6. LPS administration elevates significantly the IL-6 plasma levels in *MORG1*^*+/+*^ animals. Systemic IL-6 levels are reduced in LPS treated *MORG1*^*+/−*^ mice in comparison to *MORG1*^*+/+*^ */LPS* mice. *N* = 6 per group. *p < 0.05, **p < 0.01. **b** LPS treatment did not affect the IFNγ plasma levels in *MORG1*^*+/−*^ mice compared with *MORG1*^*+/+*^ mice indicating that the suppression of IL-6 is not an unspecific effect. N = 6 per group

Since it has already been shown that plasma cytokine levels of IL-6 correlate with survival as well as endotoxin levels in patients with septic syndrome [29], we measured the plasma concentrations of IL-6 and IFNγ. We found that LPS administration sharply elevated the plasma levels of IL-6 (*p* < 0.05) in *MORG1*^*+/+*^ mice (Fig. 5a) as previously shown [29]. However, the IL-6 plasma concentrations in endotoxemic *MORG1*^*+/−*^ mice were significantly (*p* < 0.05) lower than in *MORG1*^*+/+*^ mice (Fig. 5a). In contrast, plasma concentrations of IFNγ were enhanced by LPS administration in both genotypes (Fig. 5b).

### LPS-induced renal TNF-α expression is significantly reduced in MORG1^+/−^ mice

**Fig. 6:**
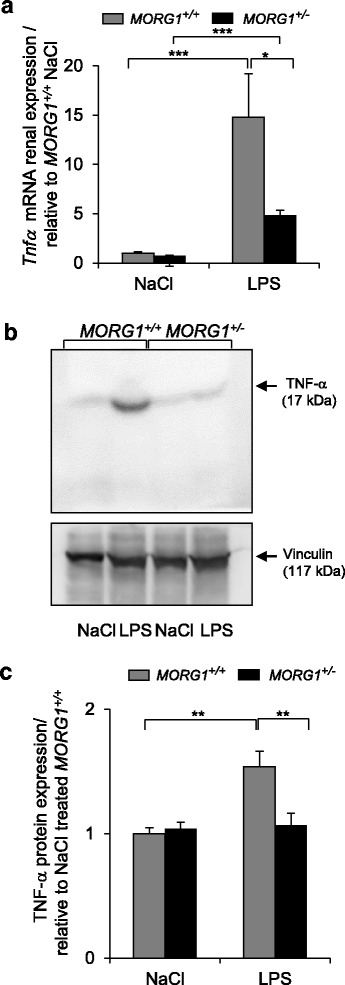
**a-c**: *Tnfα* mRNA expression in renal tissue. **a** Influence of LPS on renal expression of *Tnfα* mRNA in *MORG1*^+/+^ and *MORG1*^*+/−*^ mice was analysed by real-time PCR 24 h post LPS application. N = 6, *MORG1*^*+/+*^ LPS vs. *MORG1*^*+/−*^ LPS treated mice, *p* = 0.003; (*MORG1*^*+/+*^ LPS vs. *MORG1*^*+/+*^ saline treated mice, *p* < 0.001), *MORG1*^*+/−*^ LPS vs. *MORG1*^*+/−*^ saline treated mice, *p* < 0.001. **p < 0.01, ***p < 0.001. **b** Detection of the protein expression of TNF-α by Western blotting using whole kidney protein lysates. The protein expression of TNF-α in LPS treated *MORG1*^*+/−*^ mice was significantly lower than in endotoxemia- exposed *MORG1*^*+/+*^ mice. Representative images are shown. Four independent experiments were conducted. *N* = 4. **c** The expression of the proteins was quantified with ImageJ. TNF-α expression levels were normalized to vinculin loading and are graphically presented relative to NaCl treated *MORG1*^*+/+*^ mice. N = 4 per group, *MORG1*^*+/+*^ LPS vs. *MORG1*^*+/+*^ saline treated mice, *p* = 0.008; *MORG1*^*+/+*^ LPS vs. *MORG1*^*+/−*^ LPS treated mice, *p* = 0.004. **p < 0.01

An important indicator of renal inflammation is the local expression of the pro-inflammatory cytokine tumour necrosis factor alpha (TNF-α). Therefore, we investigated the protein and gene expression of TNF-α in renal tissue lysates after exposure to LPS. The renal mRNA expression *of Tnf-α* was strongly up-regulated in *MORG1*^*+/+*^ and *MORG1*^*+/−*^ mice 24 h post LPS administration, relative to the corresponding controls. However, we found a significantly (*p* = 0.003) lower *Tnf-α* mRNA level in *MORG1*^*+/−*^ mice after LPS application (Fig. 6a) in comparison to the endotoxemic *MORG1*^*+/+*^ litter-mates (Fig. 6a). Furthermore, Western blot analysis revealed that LPS significantly (*p* = 0.008) elevated as well the renal protein expression of TNF-α in *MORG1*^*+/+*^ mice, compared with untreated mice (Fig. 6b, c). However, the TNF-α protein levels were significantly (*p* = 0.004) reduced in *MORG1*^*+/−*^ heterozygous mice exposed to LPS in comparison to their endotoxemic *MORG1*^*+/+*^ litter-mates (Fig. 6b, c).

### MORG1 suppression affects NF-κB signaling

**Fig. 7:**
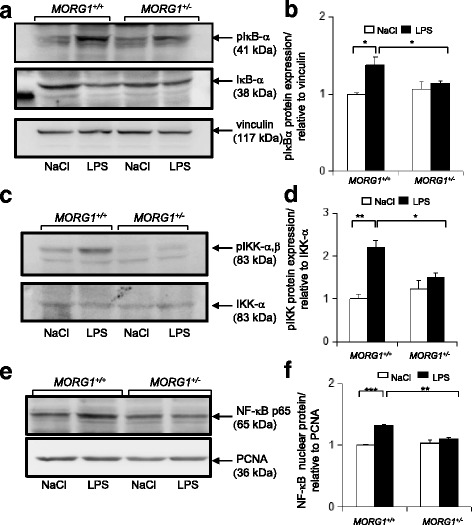
**a-f**: Analyses of NF-κB signalling in endotoxemia mice. **a** Assessment of IκB-α phosphorylation in renal tissue 24 h after induction of endotoxemia or saline. Phospho-IκB-α levels in total kidney protein lysate were detected by Western blot analyses. The expression of IκB-α is also shown. Equal loading was detected by analyses of the vinculin protein expression. Representative images are shown. Four independent experiments were performed. **b** The expression of the proteins was analysed by ImageJ software. pIκB-α expression levels were normalised to vinculin loading and are graphically presented. N = 4 per group, *MORG1*^*+/+*^ LPS vs. *MORG1*^*+/+*^ saline treated mice, *p* = 0.024; *MORG1*^*+/+*^ LPS vs. *MORG1*^*+/−*^ LPS treated mice, *p* = 0.048**.** *p < 0.05. **c** Detection of IKKα,β phosphorylation in renal tissue. Phospho-IKK-α,β levels in total kidney protein lysate was detected by Western blot. Equal loading was monitored by detection of the vinculin protein expression. Representative images are shown. *N* = 4. **d** The expression of the proteins was analysed by ImageJ software. pIKK-α,β expression levels were normalized to IKK-α expression and are graphically presented relative to NaCl treated *MORG1*^*+/+*^ mice. *N* = 4 per group, *MORG1*^*+/+*^ LPS vs. *MORG1*^*+/−*^ LPS treated mice, *p* < 0.05, *MORG1*^*+/+*^ LPS vs. *MORG1*^*+/+*^ saline treated mice, *p* < 0.01. *p < 0.05, **p < 0.01. **e** Detection of NF-κB (p65) protein in the nuclear fraction of kidney lysates of NaCl and LPS injected *MORG1*^*+/+*^ and *MORG1*^*+/−*^ mice by Western blot. Equal loading was monitored by assessment of the PCNA nuclear protein expression. Representative images are shown. Three independent experiments were performed. *N* = 3. **f** The expression of the proteins was analysed by ImageJ software. NF-κB nuclear levels were normalised to PCNA expression and are graphically presented relative to NaCl treated MORG1^+/+^ mice. *N* = 3 per group, *MORG1*^*+/+*^ LPS vs. *MORG1*^*+/−*^ LPS treated mice *p* = 0.002; *MORG1*^*+/+*^ LPS vs. *MORG1*^*+/+*^ saline treated mice, *p* < 0.001. **p < 0.01, ***p < 0.001

Since IL-6 plasma levels and TNF-α renal expression were reduced in endotoxemic *MORG1*^*+/−*^ animals after LPS application, despite the increased HIF-2α accumulation in renal tissue and as both are also regulated by the transcriptional activity of the nuclear factor-kappa B (NF-κB), we additionally assessed the activation of NF-κB by analysing the phosphorylation rate of the inhibitor of κB (IκB-α) by Western blot. We detected an LPS-dependent significant (*p* = 0.048) elevation of phospho-IκB-α (pIκB-α) levels in the *MORG1*^*+/+*^ mice compared to the *MORG1*^*+/−*^ LPS exposed mice (Fig.7a, b). Supporting this observation, the levels of the IκB-α protein were reduced in the protein lysates with higher pIκB-α content as a likely result of the IκB-α proteasomal degradation. Moreover, analyses of the IKK-phosphorylation, the upstream kinase involved in the phosphorylaton of the IκB-α, further confirmed that LPS administration elevated the levels of phospho-IKK α,β in *the MORG1*^*+/+*^ (*p* < 0.01) but not in the *MORG1*^*+/−*^ animals (Fig. 7c, d). We further explored NF-κB activation by examining protein amounts of NF-κB in the nuclear fraction of the kidney lysates. The protein levels of NF-κB were increased in the LPS-treated *MORG1*^*+/+*^ mice compared with the endotoxemic *MORG1*^*+/−*^ litter-mates (*p* = 0.002) (Fig. 7e, f). Moreover, we assayed the mRNA expression of the NF-κB target gene *iNOS* (inducible nitric oxide synthase) and our results showed a significantly lower expression of *iNOS* mRNA in the *MORG1*^*+/−*^ LPS treated mice compared with the wild-type endotoxemic mice (see Additional file 1: Figure S1), thus supporting the reduced NF-κB activation in endotoxin treated *MORG1*^*+/−*^ mice.

### Septic *MORG1*^*+/−*^ mice are protected from inflammatory T-cell infiltration and apoptosis

**Fig. 8:**
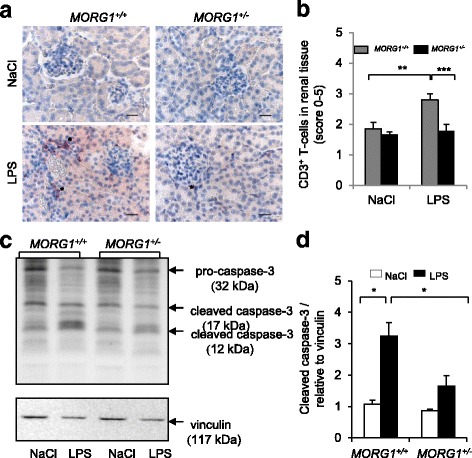
**a-d**: Reduced MORG1 expression ameliorates the peritubular infiltration of inflammatory T-cells in renal tissue and reduced renal caspae-3 cleavage in endotoxemic mice 24 h post LPS application. **a** Immunohistological detection of CD3^+^ cells. Representative images are shown. The CD3^+^ cells are shown with asterisk on the images. The nuclei were counter-stained with DAPI. The Original magnification 400×. Bars correspond to 20 μm. N = 6 per group. **b** Evaluation of CD3^+^ expression by scoring method. *N* = 6 per group, *MORG1*^*+/+*^ LPS vs. *MORG1*^*+/+*^ saline treated mice, *p =* 0.003; *MORG1*^*+/+*^ LPS vs. *MORG1*^*+/−*^ LPS treated mice, *p* < 0.001. **p < 0.01, ***p < 0.001. **c** Analysis of caspase-3 activation by Western blot in kidney lysates cytosolic fraction of NaCl and LPS injected *MORG1*^*+/+*^ and *MORG1*^*+/−*^ animals. Activation of caspase-3 was revealed by the presence of the 17 and 12 kDa protein bands, corresponding to the cleaved (activated) caspase-3. Equal loading was controlled by vinculin expression. Representative images from three independent experiments are shown. *N* = 3. **d** The proteins levels of cleaved caspase-3 were measured by ImageJ software, normalized to vinculin expression and are graphically presented relative to NaCl treated *MORG1*^*+/+*^ mice. *N* = 3 per group, *MORG1*^*+/+*^ LPS vs. *MORG1*^*+/+*^ saline treated mice, *p* = 0.002; *MORG1*^*+/+*^ LPS vs. *MORG1*^*+/−*^ LPS treated mice, *p* = 0.004. **p < 0.01, ***p < 0.001

To examine further the effects of reduced *MORG1* on renal inflammation, we assessed the renal infiltration of inflammatory CD3^+^ positive T-cells 24 h post LPS application via immunohistochemistry. We observed significantly (*p* < 0.001) less CD3 immunoreactive cells in kidney sections of the LPS exposed *MORG1*^*+/−*^ mice compared with identically treated *MORG1*^*+/+*^ animals (Fig. 8a, b). Activated, cleaved caspase-3, was investigated by Western blot from whole kidney protein lysate. Endotoxemia induced a significantly (*p* = 0.002) higher caspase-3 cleavage in wild-type mice then in saline injected wild-type mice. In contrary, reduced expression of *MORG1* in heterozygous mice attenuated the renal caspase-3 activity. We detected a significantly (*p* = 0.004) less active caspase-3 in renal lysates of endotoxemic *MORG1*^*+/−*^ compared with *MORG1*^*+/+*^ mice injected with LPS (Fig. 8c, d).

### Survival is improved in septic MORG1^+/−^ mice

**Fig. 9:**
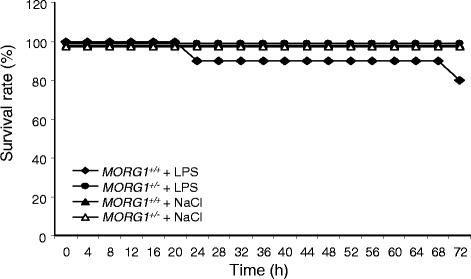
Survival studies. The animals were randomly separated into 4 groups: *MORG1*^*+/+*^ and *MORG1*^*+/−*^ injected with 0.9% NaCl, and *MORG1*^*+/+*^ and *MORG1*^*+/−*^ injected with 5 mg/kg BW LPS. The survival rate was analysed over a period of 72 h. 10 mice per group were approved by the Local Animal Committee of Thuringia and were subjected to the survival analyses. The survival rate in *MORG1*^*+/+*^ animals treated with LPS was reduced by 20% compared with the NaCl controls, whereas all *MORG1*^*+/−*^ mice injected with LPS survived the 72-h observation period

Finally, we studied mice survival in both genotypes subsequent to LPS injection. Mice were inspected every 6-h over a 72-h period. The assay was essential to the study since the *MORG1*^*+/−*^ mice were never subjected to survival analyses before. The health status of the animals was evaluated by assessment of the Clinical Severity Score (CSS) [18]. The CSS during the survival analyses of the LPS treated mice is presented in Additional file 2: Table S1 for wild-type *MORG1*^*+/+*^ and in Additional file 3: Table S2 for the heterozygous *MORG1*^*+/−*^ mice (see Additional files 2 and 3 respectively). The control groups from both genotypes injected with saline did not show signs of illness. For all of them the CSS was equal to 1 during the overall 72 h period of the survival analyses therefore the data are not presented as tables. The CSS analyses during the survival study revealed that the endotoxemic *MORG1*^*+/−*^ mice are significantly more healthy than the equally treated wild-type *MORG1*^*+/+*^ litter-mates (see Additional file 4: Figure S2). In agreement with these findings and the less impaired renal morphology and overall reduced inflammation found in the LPS-challenged *MORG1*^*+/−*^ mice, all septic *MORG1*^*+/−*^ mice survived the 72-h LPS exposure (Fig. 9). On the other hand up to 20% of the wild-type *MORG1*^*+/+*^ mice died after LPS treatment (Fig. 9).

## Discussion

Suppression of inflammation and improvement of tissue hypoxia are important factors to reduce organ injury [30]. Accelerated renal hypoxia is a key factor in the renal pathogenesis of AKI. Bacterial infection and local hypoxia oft co-exist in acute and chronic clinical conditions resulting in adverse clinical outcomes [31]. Hypoxia-inducible factors (HIFs) are the main transcription factors that regulate adaptive responses against hypoxia by activation of the expression of several target genes [32]. HIFs are rapidly eliminated in normoxic conditions due to the prolyl-hydroxylation activity of the PHDs. Yet, during hypoxia the enzymatic activity of PHDs is suppressed due to low oxygen levels leading to subsequent stabilisation and activation of HIFs [13]. There is a growing body of research demonstrating the beneficial effects of pharmacological HIF activation [7, 8, 33–35] or administration of the proteins which are generated due to HIF target gene expression such as EPO [9, 36] and VEGF. Yet, studies have consistently shown the complexity in determining the most optimal timing for application of HIF inhibitors. HIF inhibitors have also been associated with elevated mortality rates and are known to have severe side-effects, despite reducing renal inflammation and improving renal function in sepsis [8]. We and others have previously shown that the MAPK organizer protein 1 (MORG1) plays a scaffold function by accommodation and coordination of multiple proteins from different cellular networks, related to the MAPK [10] and/or PHD3/HIF axes [11]. The exact mechanism by which MORG1 interacts with all these molecules and coordinates their function is currently incompletely understood. Recent research in our laboratory has shown that MORG1 heterozygous deficiency is renoprotective in a model of renal ischemia/reperfusion due to an elevated expression of HIF [12], or partially attenuated ischemic brain injury [37] in a model of focal cerebral ischemia. Moreover, *MORG1*^*+/−*^ mice have attenuated renal damage in a model of short-time induced hypoxia in mice [14]. In this regard in the present study we focussed on the question whether a reduced MORG1 expression/ increased HIF stabilisation could potentially aid in preventing inflammation related renal injury. We utilised the well-established murine model of LPS-induced endotoxemia. In agreement with previous reports, we found that LPS administration in wild-type mice induced renal damage, mainly localized in the cortex and manifested by an increased tubular dilatation [9, 38, 39]. Although the application of 5 mg/kg BW LPS induced a mild, but not severe damage, as higher doses of LPS administration, we detected nephrotoxicity (tubular damage characterised with an induced KIM1 expression in the injured proximal tubili), although it was not so obvious as in other models [8]. On the other hand, LPS-treated *MORG1*^*+/−*^ heterozygous mice were clearly protected from tubular damage, showed a lower KIM1 tubular immunoreactivity, revealed less proteinuria and NGAL renal expression. We have to admit that although, our experimental data showed a significant histological improvement of the renal tissues in endotoxemic *MORG1*^*+/−*^ mice relatively to the wild-type *MORG1*^*+/+*^ LPS treated animals, there were still increased levels of plasma BUN in both genotypes. This discrepancy could be due to the lower sensitivity of the heterozygous mice to LPS induced nephrotoxicity, which also reflected the lower renal inflammation (a conclusion that seems not likely because *MORG1*^*+/−*^ mice are not totally protected from damage). We believe that other extrarenal factors such as volume status, catabolism may have affected the plasma BUN levels. More detailed studies including more parameters for assessment of the renal function at different time-points are necessary to proof whether the clearly observed improvement in histological injury may ultimately be also seen functionally.

The improved histological renal injury could be also related to an elevated HIF-2α protein expression in tubules (not only in nuclei, but also in the cytoplasm), which was associated with a reduced basal PHD3 protein expression in the *MORG1*^*+/−*^ compared to *MORG1*^*+/+*^ mice. LPS application further attenuated the PHD3 expression in renal tissue 24 h post endotoxemia induction in both genotypes.

Several studies have reported that PHD3 is a HIF-2α target gene, therefore one could expect an up-regulation of PHD3 in the renal tissues when HIF-2α levels are elevated, it is possible that we did not detect this because it will happen in a later time in the renal tissue. Or it is possible that in those conditions PHD3 is regulated via HIF-α independent mechanism. Furthermore, our data confirmed that endotoxemia increased the plasma levels of the pro-inflammatory cytokine IL-6 in plasma and the TNF-α renal expression in wild type *MORG1*^*+/+*^ mice [29, 40] while in contrast IL-6 and TNF-α induction were significantly reduced in *MORG1*^*+/−*^ mice. This finding open the question whether MORG1 heterozygosity could play a role not only in the HIFs pathway, but as well in the NF-κB signalling path, that is activated through LPS/ TLR4 signalling. Upon LPS or TNF-α stimulation NF-kB is activated in an IKKα,β, depended manner, which phosphorylates IκB-α leading to proteasomal degradation of IκB-α and subsequent liberation, nuclear translocation and activation of the NF-κB complex [41–43]. Indeed, we were able to detect an intact NF-κB signalling path in wild-type LPS treated *MORG1*^*+/+*^ mice, characterised with an increased phosphorylation levels of IκB-α and IKKα,β, as well as elevated NF-κB nuclear translocation and increased *iNOS* expression. Intriguingly, in endotoxemic *MORG1*^*+/−*^ mice the NF-κB signalling was impaired as the phosphorylation levels of IκB-α and IKKα,β were significantly inhibited in endotoxemic *MORG1*^*+/−*^ mice, compared with the wild-type LPS treated mice, similarly NF-κB activity was inhibited as revealed by the reduced *iNOS* mRNA production. What causes these effects in endotoxin treated *MORG1*^*+/−*^ mice? An alternative interpretation of the data could be that MORG1 heterozygous mice are less sensitive to the LPS induced nephrotoxicity, which could be associated with lower levels of TLR4 in those animals leading to reduced inflammation and an inhibition of the NF-κB signalling path compared with the wild-type endotoxemic mice. WE are currently further study this possibility, but have not answer yet. Furthermore, a recent finding of the Huh, H. et al. [44] reporting a scaffolding role of STRAP (Serine-threonine kinase receptor associated protein) for NF-κB [44]. Interestingly, STRAP is a scaffold protein, which as MORG1 belongs to the WD-40 repeats proteins and its depletion shows striking similarities with our data in regard to NF-κB regulation.

In addition, the observed anti-inflammatory effect of MORG1 down-regulation could be also related to a reduced vasodilataion and hypotension in MORG1 endotoxemic mice. Although we did not performed experiments to test this opportunity it was shown in LPS treated rats that a reduction of NF-κB signalling path was associated with an increased vasoconstriction in mice [45, 46].

One the other hand, a role of PHD3 in controlling the NF-κB signalling through inhibition of IKKβ phosphorylation, independent of PHD3 hydroxylation activity was suggested [47]. Our data also confirm this novel molecular link. These mechanisms most likely play a role in fine-tuning the regulation and coordination of HIFs (hypoxia) and NF-κB (inflammation) in the cell, involving MORG1 in a scaffolding function in the PHD3 and NF-kB complexes. As the LPS-treated mice in our experiments demonstrated a reduced expression of MORG1 (only) in the heterozygous *MORG1*^*+/−*^ mice, perhaps this strongly suggests that MORG1 may be a new piece of the NF-κB puzzle. Reduced inflammation in *MORG1*^*+/−*^ endotoxemic mice is/may also related to an inhibition of the NF-κB signalling complex. These *MORG1*^*+/−*^ anti-inflammatory effects observed in endotexemia are uncoupled from the plasma accumulation of NGAL or INFγ, where no differences were found between both genotypes after administration of LPS. On the other hand, we found that the renal expression of *ngal* and the urinary levels of NGAL were reduced in *MORG1* heterozygous mice that underwent endotoxemia treatment. Thus, the plasma NGAL protein could be a result of other organ damage and a response to a systemic effect due to inflammation, than an early marker of the renal injury. Moreover, it is difficult to use as an early marker, protein which is almost not produced in the healthy mice and humans, thus there is not a good comparison start point for the analyses.

Thus, modulation of MORG1 scaffolding function or expression, according to our data, may offer a very promising therapeutic target to help prevent acute renal injury.

## Conclusions

Our findings suggest that a modulation of the MORG1 expression ameliorates histological renal damage and has anti-inflammatory effects in a murine endotoxemia model through modulation of HIF stabilisation and/or simultaneous inhibition of the NF-κB signalling. The exact mechanism(s) of how MORG1 regulates the interplay between HIFs and NF-κB paths need further studies. Thus, here for the first time we show that MORG1 scaffold could represent an important link between innate immunity and inflammation, a finding which could be of therapeutic interest to attenuate acute renal damage related to sepsis or other inflammatory renal diseases.

## Additional files

Additional file 1: Figure S1. *iNOS* mRNA expression in endotoxemic renal tissue. Real-time PCR was performed using total kidney cDNA from saline or LPS treated wild-type respectively *MORG1* heterozygous mice. The animals underwent LPS or saline treatment for 24 h. The application of LPS significantly induced the renal *iNOS (inducible Nitric Oxide Synthase)* gene expression in both genotypes. While endotoxemic wild-type mice were characterised with a robust expression of *iNOS*, the *MORG1*^*+/−*^ mice showed only a mild activation of the *iNOS* expression. The mRNA expression ratio is presented in folds relative to the wild-type NaCl treated mice. *MORG1*^*+/−*^*/LPS* mice v.s. *MORG1*^*+/+*^ /LPS mice, ***p < 0.001. NaCl treated *MORG1*^*+/+*^ mice v.s. *MORG1*^*+/+*^ */*LPS mice, **p = 0.006. NaCl treated *MORG1*^*+/−*^ mice v.s *MORG1*^*+/−*^ /LPS mice, **p = 0.006. *N* = 4 mice per group for wild-type and *MORG1*^*+/−*^ NaCl treated mice; *N* = 7 mice per group for *MORG1*^*+/+*^ /LPS and *MORG1*^*+/−*^ /LPS mice. Data are presented as mean ± SEM. (PPTX 68 kb)

Additional file 2: Table S1. Evaluation of the animal well-being during the survival analyses of the wild-type *MORG1*^*+/+*^ mice treated with LPS by Clinical Severity Score. 10 animals were subjected to the survival analyses and were monitored every 4 h during the 72 h survival analyses. The CSS for each individual animal subjected to the survival analyses is presented in the table. The score 5 means death of the animal and is marked with asterisk on the table. (PPTX 69 kb)

Additional file 3: Table S2. Evaluation of the animal well-being during the survival analyses of the heterozygous *MORG1*^*+/−*^ mice treated with LPS by Clinical Severity Score. 10 animals were subjected to the survival analyses and were monitored every 4 h during the 72 h survival analyses. The CSS for each individual animal subjected to the survival analyses is presented in the table. All animals survived the 72 h observation period. (PPTX 69 kb)

Additional file 4: Figure S2. Graphical presentation of the Clinical Severity Score (CSS) of the four experimental groups subjected to survival analyses. 10 mice per group were monitored every 4 h during the 72 h survival analyses. The CSS assessment depicted that the endotoxemic wild-type animals have a reduced clinical health compared with the *MORG1*^*+/−*^*/LPS* mice. *MORG1*^*+/+*^*/*NaCl mice v.s. *MORG1*^*+/+*^ /LPS mice, ***p < 0.001; *MORG1*^*+/−*^*/*NaCl mice v.s. *MORG1*^*+/−*^ /LPS mice, ***p < 0.001; *MORG1*^*+/−*^*/LPS* mice v.s. *MORG1*^*+/+*^ /LPS mice, *p = 0.017. The data are presented as mean ± SEM. (PPTX 63 kb)

## Abbreviations

BW: Body weight
ELISA: Enzyme-linked immunosorbent assay
HIFs: Hypoxia inducible factors
IFNγ: Interferon gamma
IKKα,β: Inhibitor of nuclear factor kappa-B kinase subunits alpha, beta
IL-6: Interleukin-6
IκBα: Nuclear factor kappa-B inhibitor alpha
LPS: Lipopolysaccharide
MAPK: Mitogen activated protein kinase
MORG1: Mitogen activated protein kinase organizer 1
NF-κB: nuclear factor kappa-B
NGAL: Neutrophil gelatinase associated lipocalin
PHD3: Prolyl hydroxylase domain-containing protein 3
TNF-α: Tumour necrosis factor alpha
