# Supplementary material for: MORG1+/− mice are protected from histological renal damage and inflammation in a murine model of endotoxemia
Source: BMC Nephrol. 2018 Feb 5;19:29. doi: 10.1186/s12882-018-0826-4 (PMC5800025; doi:10.1186/s12882-018-0826-4)
Supplement: Supplementary file 1 — iNOS mRNA expression in endotoxemic renal tissue. Real-time PCR was performed using total kidney cDNA from saline or LPS treated wild-type respectively MORG1 heterozygous mice. The animals underwent LPS or saline treatment for 24 h. The application of LPS significantly induced the renal iNOS (inducible Nitric Oxide Synthase) gene expression in both genotypes. While endotoxemic wild-type mice were characterised with a robust expression of iNOS, the MORG1+/− mice showed only a mild activation of the iNOS expression. The mRNA expression ratio is presented in folds relative to the wild-type NaCl treated mice. MORG1+/−/LPS mice v.s. MORG1+/+ /LPS mice, ***p < 0.001. NaCl treated MORG1+/+ mice v.s. MORG1+/+ /LPS mice, **p = 0.006. NaCl treated MORG1+/− mice v.s MORG1+/− /LPS mice, **p = 0.006. N = 4 mice per group for wild-type and MORG1+/− NaCl treated mice; N = 7 mice per group for MORG1+/+ /LPS and MORG1+/− /LPS mice. Data are presented as mean ± SEM. (PPTX 68 kb) [file 12882_2018_826_MOESM1_ESM.pptx]

## Slide 1
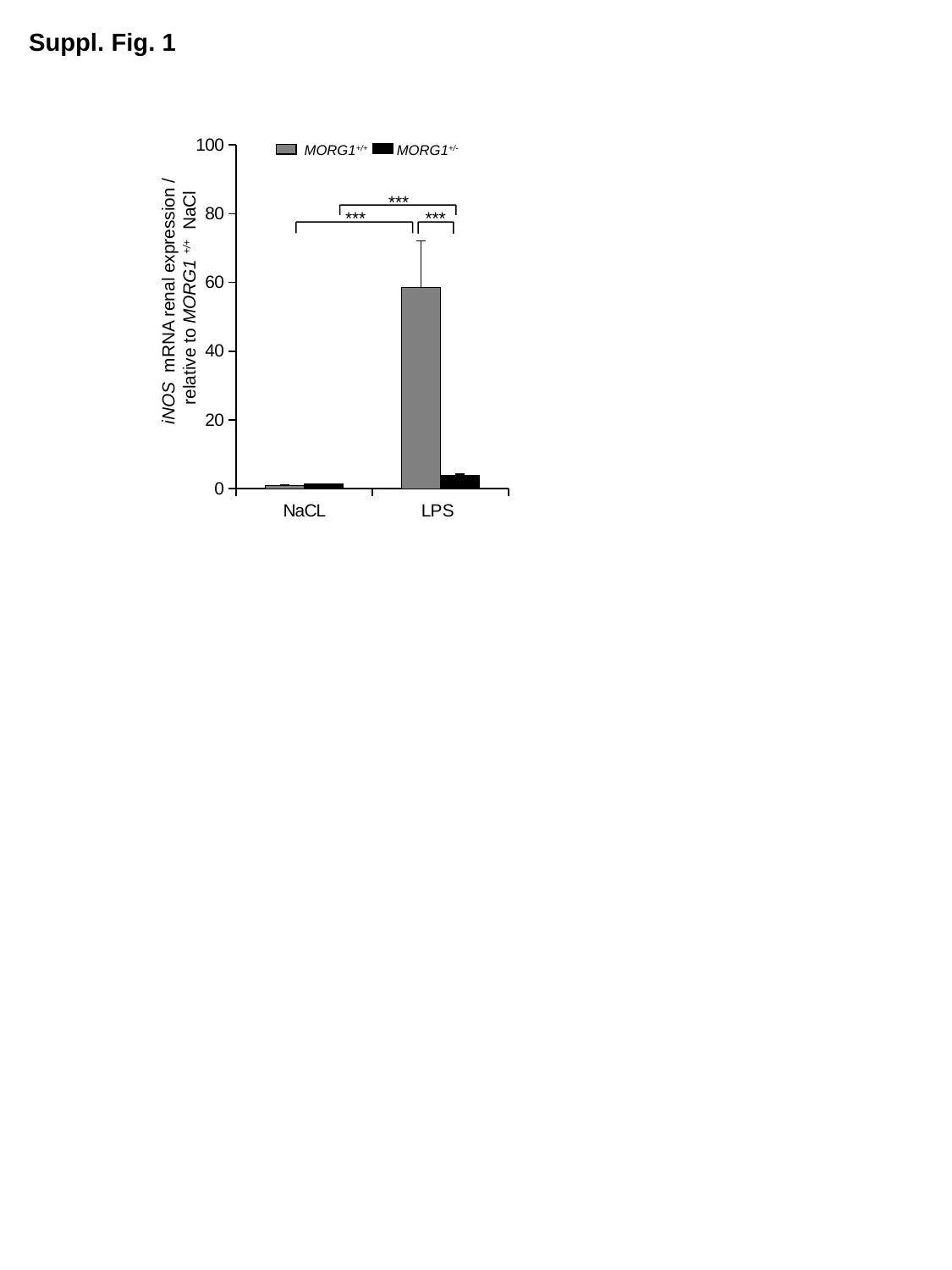

Suppl. Fig. 1
### Chart
| Category | Wt | Morg HZ |
|---|---|---|
| NaCL | 1.026 | 1.36 |
| LPS | 58.56 | 3.751 |MORG1+/+
MORG1+/-
***
***
***
 iNOS mRNA renal expression /
 relative to MORG1 +/+ NaCl
