# Supplementary material for: MORG1+/− mice are protected from histological renal damage and inflammation in a murine model of endotoxemia
Source: BMC Nephrol. 2018 Feb 5;19:29. doi: 10.1186/s12882-018-0826-4 (PMC5800025; doi:10.1186/s12882-018-0826-4)
Supplement: Supplementary file 3 — Evaluation of the animal well-being during the survival analyses of the heterozygous MORG1+/− mice treated with LPS by Clinical Severity Score. 10 animals were subjected to the survival analyses and were monitored every 4 h during the 72 h survival analyses. The CSS for each individual animal subjected to the survival analyses is presented in the table. All animals survived the 72 h observation period. (PPTX 69 kb) [file 12882_2018_826_MOESM3_ESM.pptx]

## Slide 1
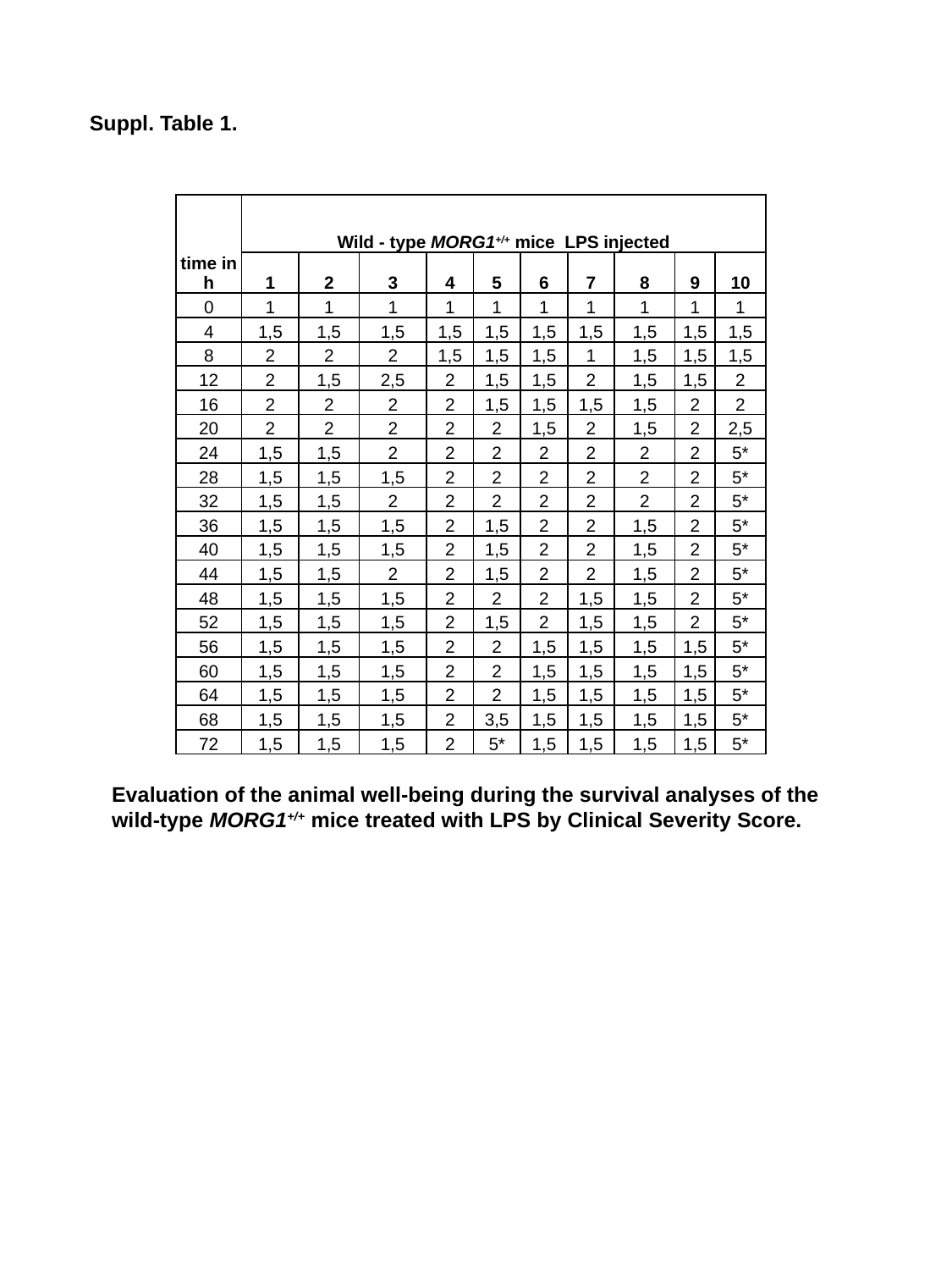

Suppl. Table 1.
| time in h | Wild - type MORG1+/+ mice LPS injected | | | | | | | | | |
| --- | --- | --- | --- | --- | --- | --- | --- | --- | --- | --- |
| | 1 | 2 | 3 | 4 | 5 | 6 | 7 | 8 | 9 | 10 |
| 0 | 1 | 1 | 1 | 1 | 1 | 1 | 1 | 1 | 1 | 1 |
| 4 | 1,5 | 1,5 | 1,5 | 1,5 | 1,5 | 1,5 | 1,5 | 1,5 | 1,5 | 1,5 |
| 8 | 2 | 2 | 2 | 1,5 | 1,5 | 1,5 | 1 | 1,5 | 1,5 | 1,5 |
| 12 | 2 | 1,5 | 2,5 | 2 | 1,5 | 1,5 | 2 | 1,5 | 1,5 | 2 |
| 16 | 2 | 2 | 2 | 2 | 1,5 | 1,5 | 1,5 | 1,5 | 2 | 2 |
| 20 | 2 | 2 | 2 | 2 | 2 | 1,5 | 2 | 1,5 | 2 | 2,5 |
| 24 | 1,5 | 1,5 | 2 | 2 | 2 | 2 | 2 | 2 | 2 | 5\* |
| 28 | 1,5 | 1,5 | 1,5 | 2 | 2 | 2 | 2 | 2 | 2 | 5\* |
| 32 | 1,5 | 1,5 | 2 | 2 | 2 | 2 | 2 | 2 | 2 | 5\* |
| 36 | 1,5 | 1,5 | 1,5 | 2 | 1,5 | 2 | 2 | 1,5 | 2 | 5\* |
| 40 | 1,5 | 1,5 | 1,5 | 2 | 1,5 | 2 | 2 | 1,5 | 2 | 5\* |
| 44 | 1,5 | 1,5 | 2 | 2 | 1,5 | 2 | 2 | 1,5 | 2 | 5\* |
| 48 | 1,5 | 1,5 | 1,5 | 2 | 2 | 2 | 1,5 | 1,5 | 2 | 5\* |
| 52 | 1,5 | 1,5 | 1,5 | 2 | 1,5 | 2 | 1,5 | 1,5 | 2 | 5\* |
| 56 | 1,5 | 1,5 | 1,5 | 2 | 2 | 1,5 | 1,5 | 1,5 | 1,5 | 5\* |
| 60 | 1,5 | 1,5 | 1,5 | 2 | 2 | 1,5 | 1,5 | 1,5 | 1,5 | 5\* |
| 64 | 1,5 | 1,5 | 1,5 | 2 | 2 | 1,5 | 1,5 | 1,5 | 1,5 | 5\* |
| 68 | 1,5 | 1,5 | 1,5 | 2 | 3,5 | 1,5 | 1,5 | 1,5 | 1,5 | 5\* |
| 72 | 1,5 | 1,5 | 1,5 | 2 | 5\* | 1,5 | 1,5 | 1,5 | 1,5 | 5\* |
Evaluation of the animal well-being during the survival analyses of the
wild-type MORG1+/+ mice treated with LPS by Clinical Severity Score.
