# Supplementary material for: MORG1+/− mice are protected from histological renal damage and inflammation in a murine model of endotoxemia
Source: BMC Nephrol. 2018 Feb 5;19:29. doi: 10.1186/s12882-018-0826-4 (PMC5800025; doi:10.1186/s12882-018-0826-4)
Supplement: Supplementary file 4 — Graphical presentation of the Clinical Severity Score (CSS) of the four experimental groups subjected to survival analyses. 10 mice per group were monitored every 4 h during the 72 h survival analyses. The CSS assessment depicted that the endotoxemic wild-type animals have a reduced clinical health compared with the MORG1+/−/LPS mice. MORG1+/+/NaCl mice v.s. MORG1+/+ /LPS mice, ***p < 0.001; MORG1+/−/NaCl mice v.s. MORG1+/− /LPS mice, ***p < 0.001; MORG1+/−/LPS mice v.s. MORG1+/+ /LPS mice, *p = 0.017. The data are presented as mean ± SEM. (PPTX 63 kb) [file 12882_2018_826_MOESM4_ESM.pptx]

## Slide 1
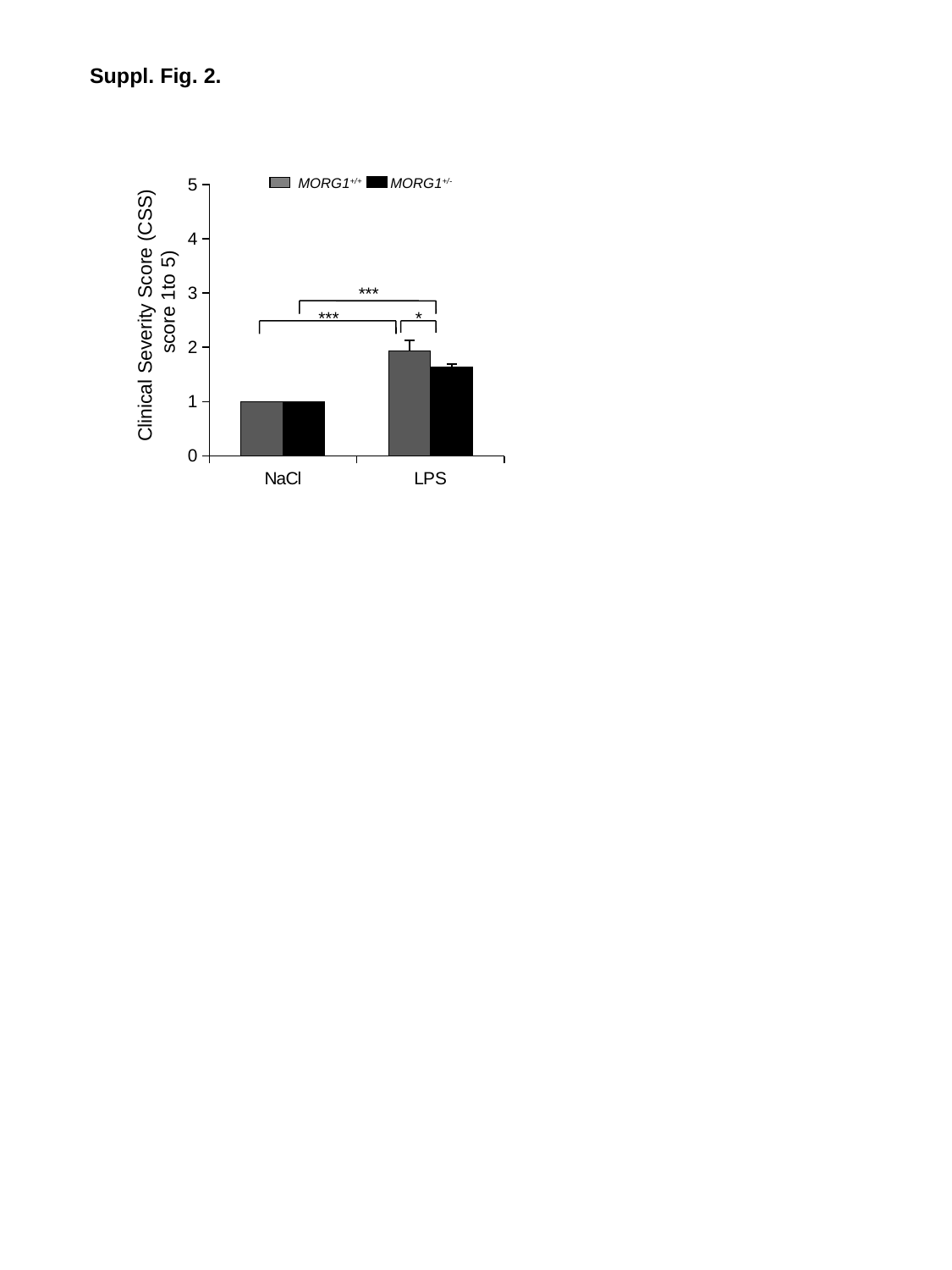

Suppl. Fig. 2.
### Chart
| Category | WT | Morg1 HZ |
|---|---|---|
| NaCl | 1.0 | 1.0 |
| LPS | 1.94 | 1.65 |MORG1+/+
MORG1+/-
***
Clinical Severity Score (CSS)
 score 1to 5)
***
*
